# Peer-Supported Diabetes Prevention Program for Turkish- and Arabic-Speaking Communities in Australia

**DOI:** 10.5402/2013/735359

**Published:** 2013-02-06

**Authors:** Nabil Sulaiman, Elaine Hadj, Amal Hussein, Doris Young

**Affiliations:** ^1^Department of Family and Community Medicine and Behavioural Sciences, College of Medicine, University of Sharjah, P.O. Box 27272, Sharjah, UAE; ^2^Department of GP, The University of Melbourne, Carlton, Melbourne, VIC 3053, Australia; ^3^Dianella Community Health, Broadmeadows, Melbourne, VIC 3047, Australia

## Abstract

In Australia, type 2 diabetes and prediabetes are more prevalent in culturally and linguistically diverse (CALD) communities than mainstream Australians. *Purpose*. To develop, implement, and evaluate culturally sensitive peer-supported diabetes education program for the prevention of type 2 diabetes in high-risk middle-aged Turkish- and Arabic-speaking people. *Methods*. A two-day training program was developed. Ten bilingual peer leaders were recruited from existing health and social networks in Melbourne and were trained by diabetes educators. Each leader recruited 10 high-risk people for developing diabetes. Questionnaires were administered, and height, weight, and waist circumference were measured at baseline and three months after the intervention. The intervention comprised two 2-hour group sessions and 30 minutes reinforcement and support telephone calls. *Results*. 94 individuals (73% women) completed the program. Three months after the program, the participants' mean body weight (before = 78.1 kg, after = 77.3; *Z* score = −3.415, *P* = 0.001) and waist circumference (*Z* = −2.569, *P* = 0.004) were reduced, their diabetes knowledge was enhanced, and lifestyle behaviours were significantly improved. *Conclusions*. A short diabetes prevention program delivered by bilingual peers was associated with improved diabetes awareness, changed lifestyle behaviour, and reduction in body weight 3 months after intervention. The findings are encouraging and should stimulate a larger control-designed study.

## 1. Introduction

Diabetes is a major and complex health problem worldwide. A recent systematic review showed that racial and ethnic minorities bear a disproportionate burden of the diabetes epidemic; they have higher prevalence rates, worse diabetes control, and higher rates of complications [1].

This is particularly true in Australia, where diabetes is rising amongst Culturally and Linguistically Diverse Communities (CALD) and particularly those from the Middle Eastern Region [2], mainly Turkish- and Arabic- speaking communities. The city of Hume in Melbourne, Australia, with a population of 148,000, has a significant proportion of people from Turkish- and Arabic- speaking countries. These communities have the highest rates for self-reported diabetes, the highest hospitalizations rate, and the second highest mortality ratio from diabetes [2–4]. Risk factors contributing to these poor outcomes identified in a recent household survey [5] include physical inactivity (76% compared to a 43% average across Victoria), a 62% prevalence of obesity, smoking in 49%, and hypercholesterolemia in 41%. These risk factors are more prevalent in Arabic-speaking community attending general practice in Sydney [6]. 

Diabetes prevention programs tailored specifically to the needs of the Turkish- and Arabic-speaking people have the potential to significantly reduce the incidence of diabetes and its impact on individuals, their families, the health care system, and community at large. Our previous work [7] conducted with these cultural groups has identified their need for more culturally appropriate information and education on lifestyle changes, coping with stresses and overcoming barriers to healthy lifestyle as a result of migration. Focus group [7] participants were also interested in partaking in a peer-supported diabetes education program. Thus, the aim of this study was to develop, implement and evaluate a culturally sensitive peer-supported education program for the prevention of type 2 diabetes in Turkish- and Arabic-speaking community in Melbourne, Australia.

## 2. Methodology

### 2.1. Research Design

The study is participatory action research. The research design is one-group Pre-test-Post-test intervention trial. 

### 2.2. Phases of Study

An expert advisory group was formed to advise on the implementation of the study, which involved three phases: (1) developing a culturally appropriate interactive peer lead diabetes prevention program (the package), (2) testing the education program in ten interactive, peer-supported, small group discussion, and (3) data collection to evaluate the program using predefined outcome measures related to physical activity and diet.

The advisory group consisted of the research team including academic general practitioners, endocrinologist, epidemiologist, dietician, diabetes educator, physiotherapist, self-management consultant and consumer representative. The team enrolled consumer representatives from the Arabic- and Turkish-Speaking communities, diabetes Australia, Victoria, relevant divisions of general practice (organized regional groups of general practices in Australia). 

The Group Education Intervention program based on the training manual is included in Table 3.

The peer leader training manual was based on guidelines and multilingual resources from the International Diabetes Institute, Diabetes Australia, and the “Better Health Self-Management program” developed by Stanford patient Education Research Center. The manual was modified based on our experience from our pilot study [7] of five Arabic- and Turkish-speaking focus groups and interviews with peer leaders. Core content and delivery of the program were determined following discussions with all stakeholders (Table 3). The intervention package comprised education on diabetes and its impact on health, risk factors and lifestyle, physical activity, and diet. The PI and project manager prepared a set of photos for relevant food items to demonstrate serving size, fat, and sugar content of the food items. The materials were translated to Arabic and Turkish languages. After completion of the package and approval of the advisory group, the peer leaders were trained on all facets of the program in two days (6-hours ×2 sessions). Leaders were paid for their training time, recruitment of participants, and implementing the program. 

Each leader recruited ten participants who attended 2 × 2 hours interactive sessions one week apart. Each group consisted of 10 participants recruited according to the criteria listed earlier. The lifestyle education intervention program was implemented and evaluated in ten interactive peer-supported small groups using pre- and postdesign. At the end of the first group discussion, each participant was given a pedometer to act as an incentive for exercise. For continuity of engagement of participants, each leader conducted a 30-minute telephone reinforcement and coaching interview with each participant one month after the second group discussion. 

The small group education intervention was based on the training manual and delivered using interactive strategies aided by education materials comprising of culturally sensitive picture booklet on Turkish and Arabic food, exercise leaflets, food basket for displaying healthy food and healthy cooking, Pedometer, drink bottles containing sugar, and the Australian Guide to Healthy Eating posters and Sugar poster.

### 2.3. Sample Selection

A media release was sent to ethnic radio stations and Arabic and Turkish print media. As a result ten bilingual peer leaders were recruited (five from the Arabic and five from the Turkish community) including ethnic workers, interpreters, health promotion workers, and teachers. The peer leaders assisted by the research team recruited group participants; these were males or females people who are 40-year old or older with one or more of the following criteria: overweight, a family history (a parent, brother, or sister) of diabetes, of Turkish or Arabic backgrounds, have had gestational diabetes, high blood pressure (140/90+), high cholesterol and/or high lipids (triglyceride), fairly inactive, or exercise fewer than three times a week. Participants were recruited through Community Health Centres and other partners, mainstream local media as well as ethno-specific radio and newspapers, community organizations and migrant resource Centres, local general practitioners, and family members of diabetic patients from the target communities. 

### 2.4. Data Collection/Analysis

Data collection was performed before and after three months intervention. Participants were requested to complete a self-administered questionnaire on the outcome indicators listed below. Peer-leaders measured participants' body weight and waist circumference before and 3-month after intervention using the same scales. Outcome indicators included changes in body weight and waist circumference, changes in knowledge and attitudes towards healthy eating, and changes in specific food shopping behaviours including dairy product, type of meats, and soft drinks.

The data was analysed using SPSS (Statistical Package for Social Sciences) for windows version 14.0. To test the differences in continuous variables between pre- and postintervention measurements, paired-samples *t*-test was used when normality was found and the non-parametric Wilcoxon Signed Ranks Test was used when normality assumption was not met. The level of significance was set at 5%. 

## 3. Results

A total of 94 subjects participated and completed the program. Women represented 73.4% (*n* = 69) of the study participants. 28% (*n* = 25) of the sample were above 55 years of age and 47% (*n* = 41) were 40–45 years old. Subjects were born in Turkey (45%), Iraq (39%), and Lebanon (12%). At baseline, half of the sample were obese (BMI ≥ 30 kg/m^2^) (Table 1). 

**Figure 1:**
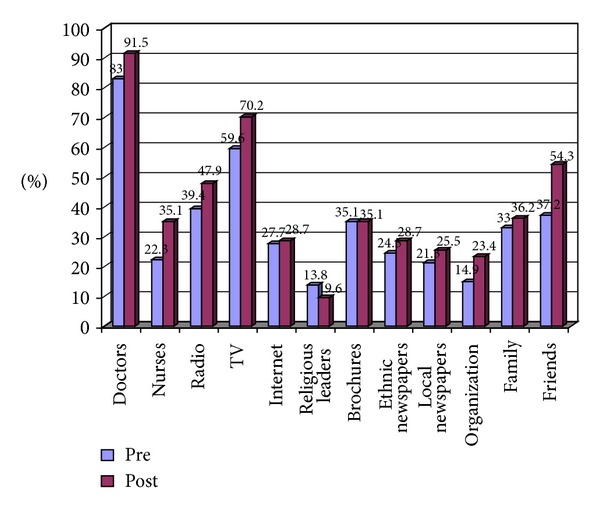
sources of health information as reported by subjects attending peer-supported self-management diabetes prevention program for Turkish- and Arabic-speaking communities of Australia (*N* = 94).

About half (*n* = 45) of the study participants reported visiting their GPs monthly, while 30% (*n* = 28) said that they visit their doctors once every six months, 7.4% (*n* = 7) yearly, and 10% (*n* = 9) reported irregular visits to their GPs.  Figure 1 shows all the sources of health information reported by participants. 

### 3.1. Weight and Waist Circumference

Significant reduction in weight (mean weight before = 78.1 kg, after = 77.3 kg; *Z* score = −3.415, *P* = 0.001) and waist circumference (mean waist before = 99.48 cm, after = 96.48, *Z* = −2.906, *P* = 0.004) was observed three months after the intervention. Reduction in weight or waist circumference did not differ significantly by gender, age, or BMI (Table 2). Similarly, participants who undertook activities to reduce their risk of diabetes did not experience more significant weight loss than those who did not (0.71 kg compared to −0.97 kg, resp., *P* = 0.597). This was also the case for waist reduction (3.30 cm compared to 7.24 cm, resp.; *P* = 0.306). 

### 3.2. Knowledge

 An understanding of the preventable nature of diabetes improved after the program from 70% to 80% (*P* = 0.055). Knowledge of cardiovascular risk factors of diabetes also increased after the intervention but was not statistically significant except for the importance of cholesterol. At baseline, 62% of the subjects identified high cholesterol as a cardiovascular risk factor compared with 77% post intervention (*P* = 0.049) (Table 2). Over half of participants stated that they are likely to get diabetes (54.8%) postintervention compared to 29.8% preintervention (*P* = 0.069). 

### 3.3. Preventive Actions against Diabetes

**Figure 2:**
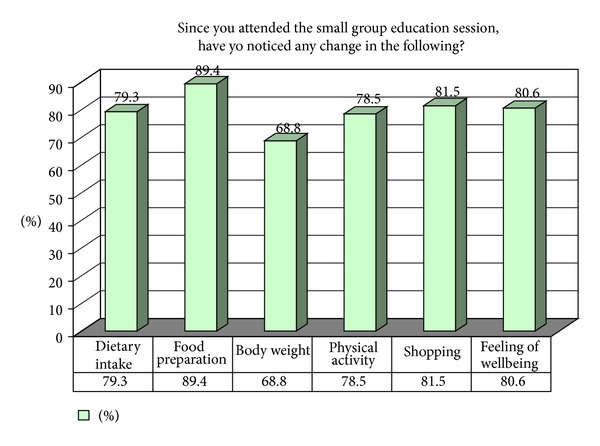
Self-reported lifestyle change postintervention as reported by subjects attending peer-supported self-management diabetes prevention program for Turkish- and Arabic-speaking communities of Australia (*N* = 94).

79.6% of participants modified their lifestyle (increased exercise and modifying their eating habits) as a result of intervention. Average time spent in walking increased significantly postintervention (*P* = 0.007). Participants who failed to take any preventative action cited lack of time to cook own meal and a preference for fast food as reasons for their attitude. The majority of participants reported changes in some aspects of lifestyle (Figure 2) including dietary habits (79%), food preparation (89%), and physical activity (79%). More than two-thirds reported reduction in bodyweight.

### 3.4. Effectiveness of the Program

Upon completion of the program, participants were asked to rate the effectiveness of peer-supported small group sessions in providing diabetes prevention on a scale from 1 (not effective at all) to 10 (very effective). The majority (68.2%) of participants (*n* = 62) found it very effective (score 9 and 10 on the scale) and 17.6% reported effectiveness of 7 and 8 on the scale. A minority (2.2%) found the program of no benefit. (3 or 4 points) and 2.2% were undecided (score of 5). The subjects identified various ways that they had gained knowledge from involvement in the peer education program including healthy eating (95%), maintaining an ideal weight (70%), means of losing weight (75%), activities and exercises to do regularly (73%), and access to information and resources (48%). Personal attitudinal change to diabetes prevention was reported by 42% of the study sample. 

## 4. Discussion

The results of this study show that a limited intervention administered by trained lay bilingual peers equipped with culturally tailored educational resources in the native language was associated with significant and lasting (three months) improvement in anthropometric measurements, knowledge, and attitudes to diabetes prevention. The outcome was an increase in exercise as well as significant modification of shopping, cooking, and eating habits by a significant majority of the participants. These findings are consistent with a similar study of community-based, peer-supported diabetes self-management program for Spanish-speaking people [8], which demonstrated all of the previously mentioned as well as a reduction in health care utilization. Similarly a recent review of diabetes prevention programs [9] concluded that didactic education was ineffective in improving diabetes control when compared to peer support. In addition, a nine-month small peer-led group education program for 54 Turkish migrants in the Netherlands [10] confirmed that ethnic-specific diabetes programs were appreciated by both patients and their general practitioners. 

A positive aspect of our program is the significant reduction in participant's weight and waist circumference after only four hours of intervention. Whether greater outcomes could be achieved by longer intervention remains to be clarified. Factors significantly related to this reduction were not identified in this study. The self-reported changes in behaviour were liable to bias. Similarly the measurements of weight and waist circumference were also prone to bias because it was conducted by the peer leaders; they would have been more valid if measured by independent assessors.

The success of our program is consistent with the conclusion of the most recent systematic review of 11 RCTs of culturally tailored diabetes education programs for ethnic minorities with type 2 diabetes living in developed countries that identified “culturally tailored health education” as more effective than “usual care” in improving blood sugar control and knowledge of diabetes [11]. Most recently, a RCT of 345 diabetics randomized to community-based peer-supported diabetes self-management program showed significant improvement in symptoms of hypoglycaemia, a reduction in the frequency of depression, improved communication with physician, healthy, eating and reading of food labels [12]. Similarly Choudhury et al. [13] reported the outcome of a peer-led education program for diabetes and cardiovascular disease in a Bangladeshi population living in the UK which demonstrated improvement in several aspects of diabetes self management.

## 5. Limitations of the Study

This study is based on self-reporting of changes in behavior; thus it is prone to bias. However, changes in weight were recorded from self-reports as well as objective weighing of participants by peer leaders and were therefore less prone to bias. Similarly changes in waist circumference were measured by peer leaders. A possible overestimation of the intervention effectiveness may be due to some characteristics of the sample such as voluntary participation, gender (mainly females), and frequent GP visits. The education program was of three months duration, and it is possible that the observed changes may not be sustainable. On the other hand, a longer program may yield more impressive results. The encouraging results obtained by our group should be confirmed by a larger controlled study, and replication of the study in other ethnic groups should be encouraged. 

## 6. Conclusion

A short-term, cost-effective diabetes prevention program comprising of two 2-hour group education sessions supported by lay bilingual peers and reinforced with telephone reminders was successful in changing lifestyle behaviour and reducing weight and waist circumference. Sustainable changes may be achieved by trained peers employed by community health services.

## Figures and Tables

**Table 1:** Characteristics of study subjects enrolled in the peer-supported self-management diabetes prevention program for Turkish- and Arabic-speaking communities of Australia (*N* = 94).

Variable	*N*	%
Gender		
Male	25	26.6
Female	69	73.4
Age		
40–45	41	46.6
46–50	19	21.6
51–55	3	3.4
56–59	12	13.6
60 and above	13	14.8
Country of birth		
Turkey	42	44.7
Lebanon	11	11.7
Iraq	37	39.4
Syria	3	3.2
Other	1	1.1
Weight mean (sd)	78.1 (14.1)	
Height mean (sd)	161 (9.8)	
Waist circumference mean (sd)	99.5 (14.2)	
BMI kg/m^2^ mean (sd)	30.3 (6.3)	
<18.5	—	—
18.5–24.9	18	19.1
25.0–29.9	29	30.9
30.0–34.9	29	30.9
≥35	18	19.1

**Table 2:** Comparing knowledge and perceived risk of getting diabetes among study subjects before and after a peer-supported self-management diabetes prevention program for Turkish- and Arabic-speaking communities of Australia.

	Before		After		*P* value
	*N*	%	*n*	%	
Knowledge					

Do you think Diabetes can be prevented?					
Yes	62	69.7	74	78.7	0.055**
What can increase a person's chance of getting diabetes?					
Overweight	76	80.9	79	84	0.690
Underweight	22	23.4	19	20.2	0.711
Having family member with diabetes	67	71.3	77	81.9	0.121
High blood pressure	51	54.3	62	66	0.090**
High cholesterol	58	61.7	72	76.6	0.049*
Physical inactivity	52	55.3	52	55.3	1.00
Eating lots of food with sugar	58	61.7	55	58.5	0.761
Giving birth to large babies	36	38.3	39	41.5	0.701
Being over 45	51	54.3	55	58.5	0.585
Stress	76	80.9	77	81.9	1.00
Smoking	45	47.9	57	60.6	0.073**
Other	9	9.6	13	13.8	0.424
What behaviours can help prevent diabetes?					
Healthy Lifestyle	80	85.1	72	76.6	0.200
Healthy Diet	77	81.9	84	89.4	0.210
More exercise	77	81.9	83	88.3	0.286
Weight control	76	80.9	79	84.0	0.664
Regular checkups	77	81.9	66	70.2	0.090**
Others	17	18.1	15	16.0	0.832

Perceived risk of getting diabetes					

How likely do you think you are to get diabetes?					
Unlikely	17	18.1	15	17.9	0.069**
Likely	28	29.8	46	54.8	
Not sure	38	40.4	23	27.4	
Why do you think you are at risk of developing diabetes?					
Overweight	56	59.6	68	72.3	0.029*
Family member with diabetes	55	58.5	67	71.3	0.050*
High blood pressure	36	38.3	46	48.9	0.154
High cholesterol	43	45.7	61	64.9	0.006*
Doing little exercise	51	54.3	57	60.6	0.451
Eating fatty food	38	40.4	46	48.9	0.256
Being under stress	53	56.4	64	68.1	0.108
Smoking	27	28.7	48	51.1	0.001*
Others	8	8.5	11	11.8	0.629

*Significant *P* value (≤0.05)

**Borderline significance (0.05 < *P* < 0.10).

**Table 3:** The group education intervention program based on the training manual.

Topic	Title	Time/min.
Session 1		

(1)	Welcome and introduction	20
(2)	Program outline	5
(3)	Prediabetes talk	25
(4)	Benefits of healthy lifestyle	10
(5)	Weight management	25
(6)	Reducing fats and sugars	25
(7)	Pedometer	5
(8)	Quiz A	5

Session 2		

(9)	Exercise session	10
(10)	Review homework from previous week	10
(11)	Physical activity	30
(12)	Healthy eating	30
(13)	Self care	10
(14)	Stress management	10
(15)	Quiz B	5
(16)	Bringing it altogether	15

Education tools		

(i) Electronic weight scale and tape measure		
(ii) Poster: the Australian guide to healthy eating for display		
(iii) Food basket for display		
(iv) Pedometer		
(v) Oil spray		
(vi) Sugar poster		
(vii) Drink bottles containing sugar		
(viii) Picture booklet: foods and exercise		
(ix) Butchers' paper, bulldog clips, blue tack		
